# Biosynthesized iron oxide-nanoparticle encapsulated hydrogel functionalized with platelet-rich plasma (PRP) accelerates wound healing in an animal model

**DOI:** 10.1039/d5na00621j

**Published:** 2025-09-11

**Authors:** Lipsa Leena Panigrahi, Siddharth Satpathy, Pallavi Samal, Shashank Shekhar, Shakti Ketan Prusty, Manoranjan Arakha

**Affiliations:** a Center for Biotechnology, Siksha ‘O’ Anusandhan (Deemed to be University) Bhubaneswar Odisha 751003 India marakha@soa.ac.in manoranjan.arakha@gmail.com; b Indian Institute of Technology Hyderabad India; c School of Pharmaceutical Sciences, Siksha ‘O’ Anusandhan (Deemed to be University) Bhubaneswar 751003 Odisha India

## Abstract

Wound healing is rendered less effective mainly due to exudate overload, bacterial growth, and limited growth factors in most cases, resulting in delayed wound healing and complications. This study reveals a new class of smart wound-healing hydrogels encapsulated with biosynthesized iron oxide nanoparticles for accelerated antimicrobial activity and wound healing. Screening these organic hybrid hydrogels revealed promising wound healing and antimicrobial properties by controlled protein secretion from the hydrogel containing PRP, alongside the mitigation of infection due to the bacteria. The hydrogel antimicrobial activity was boosted *via* green-synthesized IONP incorporation, illustrating more pronounced killing in Gram-negative bacteria, which is highly consistent with TEM-morphological alterations in the bacteria's structure, cell wall, and membrane. The chitosan-based hydrogel exhibited the lowest half-maximal scavenging concentration. The hydrogels also exhibited high cell viability and growth. Further investigation into the wound healing activity of the hydrogel was conducted using an animal model, which showed healing in 18 days compared to the control and standard. Overall, this study demonstrates a feasible design for tailoring new surface-functionalized organic-inorganic hybrid hydrogels as promising antimicrobial and wound healing agents.

## Abstract 2

The integration of biosynthesized iron oxide nanoparticles (M-IONPs) within a platelet-rich plasma (PRP)-functionalized hydrogel presents a novel bioactive platform for accelerated wound healing.

## Graphical abstract

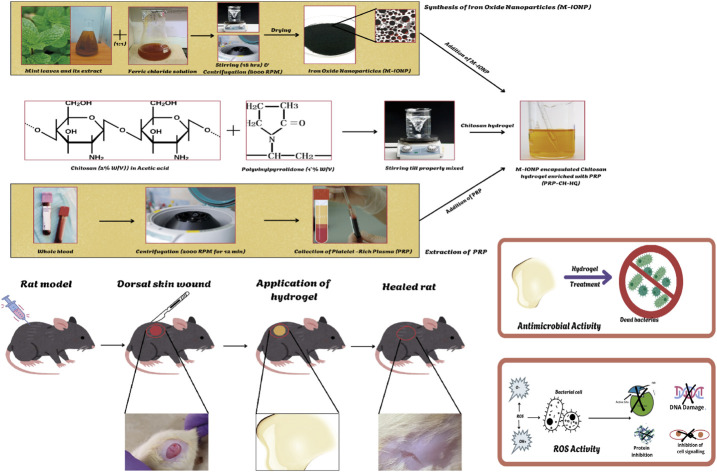

## 1. Introduction

Wound healing therapies need to become more efficient, robust, and quick as post-surgery sores, burns, skin and cancer-related ulcers, and diabetic foot ulcers increase steeply. Wound healing occurs in 3 phases: inflammation, proliferation, and remodelling.^1^ Traditional and current healing strategies, such as dressing wounds with polymeric bandages, plasters, and cotton wools and applying antibiotic-loaded polymers and epithelial autografts, are very time-consuming and cumbersome processes that sometimes become disruptive.^2,3^ Additionally, these methods are very costly, burdening patients financially. Recently, hydrogels have been extensively employed for wound dressing purposes due to their myriad benefits such as facilitating a moist environment for enhanced granule tissue development and epithelium growth surrounding the wound, preventing dehydration of the wound area, increasing oxygen availability, absorbing wound exudate, and increasing cell migration and tissue regeneration.^2,3^ However, the potential of hydrogels for biomedical applications is hugely limited due to their poor surface adhesion and mechanical properties, bacterial proliferation, and exudate accumulation.^3^

PRP is derived from blood containing concentrated platelets and is extensively utilized in regenerative medicine, which has currently been advanced for clinical usage.^4^ When platelets are activated by calcium/thrombin, their degranulation begins leading to the secretion of elevated concentrations of multiple GFs. The formation of gel is facilitated by the high amount of fibrinogen present in the plasma, which forms polymeric fibrin networks and binds to GFs to ameliorate clinical efficacy.^5^ Moreover, the lack of efficient mechanical strength renders PRP gel less effective in curing irregular wounds.^6^ The major challenge associated with PRP gel is the susceptible breakdown and rapid clearance of GFs after being administered in a protease-rich wound environment.^6,7^ Moreover, the limited production of such blood-derived products requires more efficient use to improve therapeutic efficiency.^7^

Nanoparticles are excellent agents that can be incorporated into these hydrogels, giving them enhanced mechanical strength and accelerating the wound-healing process.^8,9^ Hydrogels synthesized from biomaterials, like gelatin (Gel), hyaluronic acid, and chitosan (CH), are quite efficient due to their biocompatibility, biodegradability, easy delivery, and proper swelling properties.^10^ The gel synthesized from collagen hydrolysis possesses a brilliant hydrogel-forming ability for wound dressing due to its outstanding water-holding and emulsification properties. Additionally, the arginine-glycine-aspartic acid (RGD) motif present in the gel allows for efficient cell adhesion and proliferation in the wound area owing to the facilitation of chemical groups for the conjugation and modification of the gel structure by the RGD motifs.^11^ Another potential hydrogel being explored by researchers recently for wound healing purposes is the chitosan (CH) hydrogel. CH hydrogel has excellent antibacterial, antioxidant, anti-inflammatory, and bioactivity properties, making it a highly effective wound-healing agent.^12^ The attachment of the CH hydrogel to the bacterial cell wall occurs *via* electrostatic interactions between the bacterial cell wall and the chitosan's amino groups.^13^ Omer *et al.* also explored CH hydrogel as a drug vehicle; they developed an aminated CH hydrogel with the help of sodium tripolyphosphate (TPP), which serves as an anionic crosslinker, and employed it to encapsulate curcumin.^14^ This hydrogel showed a slow and controlled release of curcumin, thereby turning out to be an excellent drug vehicle.^14^ Recently, the incorporation of growth factors into hydrogels has been studied for wound healing processes. Santhini *et al.* developed a self-assembling peptide nanohydrogel and incorporated platelet-derived growth factor-BB (PDGF-BB), which showed sustained release of PDGF-BB for 48 h and resulted in 99.5% wound healing in an animal model on day 21.^15^ Similarly, a printable gelatin methacryloyl (GelMA) hydrogel scaffold was encapsulated with vascular endothelial growth factor (VEGF), and its application in the porcine wound model showed sustained release of VEGF and increased endothelial cell migration.^16^

Owing to the benefits of PRP, we aim to design a hybrid of hydrogel and PRP, which may enable PRP to elicit its enhanced therapeutic potential in clinical use. Currently, chitosan is emerging as an effective base for hydrogel. It is a natural polysaccharide and is approved by the Food and Drug Administration (FDA) owing to its commercial utilization in wound dressings, such as Flaminal Hydro Alginate Gel (Flen Health UK, Ltd) and Purilon Gel (Coloplast Ltd.).^17,18^ Additionally, chitosan can form cross-linked networks with PVP (polyvinylpyrrolidone) through hydrogen bonding.^19^ The amine (–NH_2_) and (–OH) groups form hydrogen bonds with the carbonyl (–C=O) in PVP, which improves the mechanical strength and stability of the hydrogel.^20^ Calcium/thrombin present in the plasma acts as a gelation mediator in producing homogeneous-penetrated networks.^21^ Due to its flexibility and structural modalities, the as-formed hydrogel-PRP composite has tremendous potential to heal wounds and offer a regulated release of PRP-derived therapeutic molecules.^22^ The integration of biosynthesized iron oxide nanoparticles (M-IONPs) with platelet-rich plasma (PRP) into a chitosan-based hydrogel establishes a multifunctional wound dressing capable of simultaneously promoting hemostasis, exerting antimicrobial effects, and enhancing tissue regeneration. The plant-mediated synthesis of M-IONPs offers significant advantages over traditional chemical methods by ensuring superior biocompatibility, minimal cytotoxicity, and eco-friendly synthesis, which are qualities often absent in conventional nanoparticle fabrication. PRP enriches the system with a natural reservoir of growth factors that synergize with the chitosan matrix to activate and sustain key wound healing pathways. Furthermore, the hydrogel's injectable form enables site-specific application and prolonged retention at the wound bed, enhancing localized therapeutic outcomes. In contrast to systems such as lignin-mediated silver nanoparticle-loaded hydrogels reported by Yu *et al.*,^23^ our biosynthetic approach uniquely combines plant-derived M-IONPs with PRP in a biopolymer framework. This comparative framework underscores the novelty and translational potential of our hydrogel system in the field of advanced wound care. Therefore, research is currently focused on encapsulating nanoparticles and PRP into biomaterials, which can increase the lifetime of PRP and allow the controlled release of growth factors from PRP. The present study is focused on the construction of a hydrogel to facilitate wound healing and dressing, which could sustain protein release from the hydrogel dressing, enhance re-epithelialization, and mitigate wound-related infections. With this motive, we designed a PRP-CH hydrogel incorporated with biosynthesized nanoparticles and loaded them into the hydrogel through the process of electrostatic interactions. Hydrogel dressings were characterized for their morphology, swelling ratio, specific surface area, porosity, and protein secretion. To ensure the safety of the hydrogel dressing formulations, antibacterial properties, antioxidant activity, and cytotoxicity studies were conducted. Finally, the wound healing process and effectiveness of the M-IONP-incorporated PRP chitosan hydrogel in wound dressings were evaluated using an *in vivo* rat model.

## 2. Materials and methods

The chemicals utilized for the experiments were of analytical grade and were used in their original state. Ferric chloride hexahydrate (FeCl_3_·6H_2_O), ferrous chloride tetrahydrate (FeCl_2_·4H_2_O), ferric chloride (FeCl_3_), sodium hydroxide (NaOH), methylene blue, ascorbic acid (Vit. C), and DPPH (2,2-diphenyl-1-picrylhydrazyl C18H12N5O6) were obtained from SRL Chemicals Pvt. Ltd, India. Sulphoxymethozole (C10H11N3O3S), brain heart infusion broth (BHIB), Bradford reagent and Congo red were purchased from Himedia. Hydrogen peroxide was purchased from Molychem Pvt. Ltd Bacterial strains of *Escherichia coli* (MTCC-443), *Staphylococcus epidermidis* (MTCC-435), and *Staphylococcus aureus* (MTCC-441) were obtained from the Institute of Microbial Technology (IMTECH), Chandigarh, India.

### 2.1. Preparation of PRP/M-IONP incorporated chitosan hydrogel

#### 2.1.1. Preparation of leaf extract and synthesis of iron oxide nanoparticles (M-IONPs)

*M. spicata* (Pudina) leaves were procured from the local market of Bhubaneshwar, Odisha, India. Fresh mint leaves were used to extract *M. spicata* leaves. The stemless leaves were thoroughly cleaned with distilled water and cut into small bits. Following a thorough washing procedure with double-distilled water, ten grams of leaves were added to 100 mL of double-distilled water for washing and heated at 80 °C. Centrifugation for 15 min at 8000 rpm was performed to remove and separate the pellet and the extract. Following centrifugation, the supernatant was meticulously removed by filtration and kept for later use in a dry, clean beaker. For the green synthesis of M-IONP, 0.01 M ferric chloride anhydrous and the mint leaf extract were taken in a 1 : 1 proportion in a clean, sterilized flask. There was an immediate color change upon adding the mint leaf extract to the ferric chloride solution. The solution was thoroughly mixed in a shaker at 35 °C overnight. Next, it was centrifuged at 8000 rpm for 15 min for separation. The pellet was further washed twice with deionized water. The pellet was collected and dried at 60 °C.

#### 2.1.2. PRP preparation

Platelet-rich plasma (PRP) was extracted using the Choukroun method.^24^ 10 mL anticoagulant-free blood was taken in a sterile vial, maintaining an aseptic environment, and immediately centrifuged at 2700 rpm for 12 min. The supernatant was carefully extracted from the test tube. The platelet concentration in platelet-rich plasma (PRP) was quantitatively assessed within 1 hour of collection using an automated hematology analyzer (Model: Sysmex XP-300, Manufacturer: Sysmex Corporation, Japan). Briefly, 50 μL of PRP was aspirated using the analyzer, and platelet counts were recorded directly in units of ×10^5^ μL^−1^. The analysis was performed in triplicate (*n* = 3), and the results were expressed as mean ± standard deviation.

#### 2.1.3. Preparation of PRP/M-IONP incorporated chitosan hydrogel

A 0.2% acetic acid solution was used to dissolve 0.6 mg chitosan (Sigma Aldrich; extra pure low molecular weight chitosan with a degree of deacetylation of min 90%) by stirring at ∼400 rpm. Then, polyvinylpyrrolidone (1% W/V) was poured into the CS hydrogel under stirring conditions, and the solution was left to stir for 1 h. Next, 0.1% (W/V) as-prepared M-IONP was mixed properly with the CS/PVP hydrogel by vigorous stirring. The hydrogel was probe-sonicated for 10 min to obtain a homogeneous composition. To encapsulate PRP onto the hydrogel, 10 mg equivalent of PRP was added to the M-IONP-hydrogel solution at 8000 rpm for 2 h (Table 1). The temperature throughout was maintained at 37 °C.

**Table 1:** Composition of PRP–CH–HG hydrogel formulation

Composition	PRP–CH–HG
Chitosan	0.02 mg mL^−1^ or 0.002% (w/v)
PVP	1% (w/v)
M-IONP	4 mg mL^−1^ or 0.4% (w/v)
PRP	0.33 mg mL^−1^ or 0.0333 (w/v)

Three hydrogels were prepared based on the highest antimicrobial activity concentration of the active component, M-IONP, and were subsequently tested for their antibacterial propensities. The hydrogel showing superior effects was further assessed to understand its wound-healing activity in an animal model. A lower polymer content was chosen to ensure that the hydrogel maintained fluid gel consistency for easy application.

For cytotoxicity, anti-inflammatory, haemolysis, and antimicrobial analysis, the hydrogel was diluted. The original dose was considered to be 100%; consequently, a series of dilutions of 50% and 25% were prepared. These were denoted as 25×, 50×, and 100×.

### 2.2. Characterization of samples

#### 2.2.1. Characterization of M-IONP

UV-visible spectroscopy was employed for the primary optical characterizations. The functional groups present on the nanoparticles were determined by a Fourier Transform Infrared spectrometer (FTIR) in transmission mode, with the wavenumber ranging from 4000 to 500 cm^−1^. The particle size and morphology were examined using a Field Emission Scanning Electron Microscope (FE-SEM).

#### 2.2.2. Characterization of the PRP–M-IONP–CH hydrogel

FE-SEM was employed to estimate the morphology of the PRP–M-IONP–CH hydrogel. For FE-SEM, a fine layer of gold coating of the hydrogel was applied to achieve conductivity in order to obtain high-resolution surface morphology imaging. A 10 kV working voltage was maintained for FE-SEM operation. The hydrodynamic size of the PRP–M-IONP–CH hydrogel was measured by employing dynamic light scattering (DLS). A 90 Plus Particle Analyzer (Cordouan Vasco3, France) instrument was utilized for the DLS experiment and the zeta potential measurement. The functional groups present on the hydrogel were determined with FTIR (BRUKER-TENOR27, Germany).

### 2.3. Property analysis of the PRP–M-IONP–CH hydrogel

#### 2.3.1. Analysis of swelling

The hydrogel's swelling ratio was determined by immersing the samples in 100 mL PBS, maintaining the pH at 7.2 and the temperature at 37 °C. Starting with the initial sample weight (*W*_0_) of 20 mg, the removal of hydrogels from PBS at different time points (5, 10, 15, 20, 25, 30, 35, 40, and 45 min) was performed, followed by proper drying with filter paper. Next, using the weight (*W*_*t*_) of these dried samples, the swelling ratio was calculated using the following equation:1
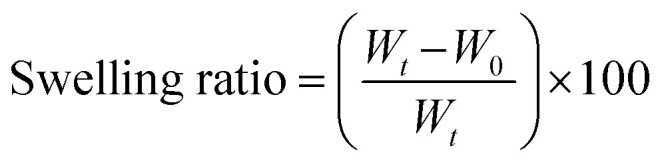


#### 2.3.2. *In vitro* degradation

Hydrogel degradation was estimated by incubating them in PBS at pH 7.2 and 37 °C for 7 days. Then, they were removed from PBS after one, three, and seven days. The hydrogels were gently washed with distilled water and blotted to remove excess surface moisture. The samples were then placed in a vacuum desiccator at room temperature (25 ± 2 °C) for 48 hours and then weighed. Eqn (2) was used to calculate the degradation percentage, where *W*_0_ is the initial weight and *W*_*t*_ is the final weight of the hydrogels. Each degradation experiment was performed in triplicate and averaged to estimate the weight loss percentage:2
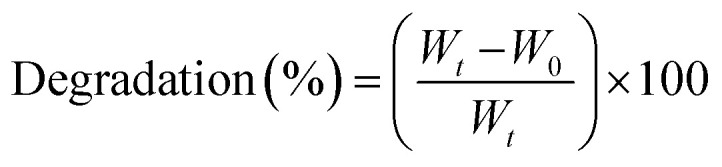


#### 2.3.3. Antioxidant assay

DPPH was used to calibrate the antioxidant properties of the produced hydrogel.^25^ In short, a methanolic DPPH stock solution containing 0.2 mM was prepared. In a 96-well microtiter plate, 100 μL of the DPPH methanolic solution was mixed with 100 μL of the hydrogel at different dilutions (25×, 50× and 100×)). As a reference, 1 mg mL^−1^ of ascorbic acid was collected. The nanoparticles were incubated for 30 min at 35 °C while being constantly shaken. At 517 nm, absorbance was measured. The following equation was used to calculate free radical scavenging activity:

3 Radical scavenging activity (%) = (1 − *A*_sample_/*A*_control_) × 100,

where *A*_sample_ is the absorbance value of the reaction mixture containing the test sample and *A*_control_ is the absorbance of the control reaction mixture containing only the radical solution without any sample.

The plot was generated between % DPPH free radical scavenging activity and hydrogel concentration.

#### 2.3.4. Spreadability of the hydrogel

The spreadability of the gels was evaluated using the parallel glass plate method, as reported in previous studies (Mutimer *et al.*, 1956; Pandey *et al.*, 2014; Sinko, 2011). The spreadability capacities of gels were assessed by placing 0.2 g of gel in a circle measuring 2 cm in diameter on a glass plate, followed by the use of a second plate. For five minutes, a weight of 200 g was allowed to lie on the upper glass plate. The circumference of the circle was measured after spreading the gel.*S* (%) = *A*/*x* × 100,where *S* = spreadability, *A* = max. spread area and *x* = total weight of the gel.

### 2.4. Biocompatibility of hydrogel

#### 2.4.1. Hemolysis assay

The hemolysis propensity of the hydrogels was determined using the absorbance technique.^26^ Whole blood (Wistar rat) was centrifuged at low speed at 1000 rpm for 10–15 min to separate the plasma. The supernatant was aspirated, leaving behind the RBC. Then, the RBC was washed by suspending them in PBS, followed by centrifugation. The hemolytic agent Triton-X was used as the positive control, while the negative control was PBS. The samples were incubated in a 96-well plate at 37 °C for 1–2 h. An absorbance reading at 450 nm specific to hemoglobin was taken. High absorbance indicated high hemolysis.

#### 2.4.2. *In vitro* anti-inflammatory assay

The *in vitro* anti-inflammatory assay was performed using the denaturation method.^27^ Briefly, various concentrations of hydrogel were prepared in the range of 5–100 μg mL^−1^, and 20 μL from them were added to a 200 μL aqueous solution of bovine serum albumin (BSA) (1% W/V) and 4.8 mL of phosphate buffer solution (PBS, pH 6.4) and mixed thoroughly. Next, their incubation at 25 °C was performed for 20 min, followed by heating at 57 °C for another 20 min. After that, the samples were cooled, and from the absorbance value of UV–Vis spectrophotometry at 660 nm wavelength, their turbidity was determined. The percentage inhibition of protein denaturation was calculated using diclofenac sodium as a standard drug.

#### 2.4.3. *In vitro* cytotoxicity analysis by MTT assay

Human embryonic kidney (HEK) cells were cultured in DMEM/F12 media, supplemented with 10% FBS and 1% penicillin. The humidity of the atmosphere was kept at 95%, and the CO_2_ concentration was kept to be 5% in a CO_2_ incubator. The washing of the culture was properly performed with PBS, followed by trypsinization with 1× trypsin–EDTA. Cell counting was performed using a hemocytometer. Cell viability was determined using the MTT assay. UV light was used to sterilize the hydrogel, and these sterile samples were pipetted into the wells of the 96-well plate. Next, 10^4^ cells per well were maintained in 50 μL of DMEM/F12 in the presence of 10% FBS and 1% antibiotics, maintaining the same cell culture conditions for the next 3 days. Three wells were kept as a negative control, which did not contain any sample. After incubating for three days, each well's medium was removed, and 100 μL of 0.5 mg mL^−1^ MTT solution was added to each well and then incubated at 37 °C. After 4 h, the supernatants were discarded, followed by the addition of 100 μL 0.1% HCl and isopropanol for the dissolution of purple crystals. The absorbance of the samples was recorded at 570 nm using an ELISA reader (Convergent EL-Reader 96×, Germany).

### 2.5. Antimicrobial propensity of the hydrogel

#### 2.5.1. *In vitro* antibacterial test

The hydrogel's antibacterial property was determined by testing its efficacy on the selected bacterial species, namely *Staphylococcus aureus* (*S. aureus*) and *Staphylococcus epidermidis* (*S. epidermidis*) as positive bacterial strains and *Escherichia coli* (*E. coli*) as a negative bacterial strain. The control sample was the one with only the culture medium, and no sample Microbiological Safety Cabinet Class II was used to perform all the bacterial and cell culture studies. The antibacterial studies were carried out in triplicate; then, the average was taken for analysis.

#### 2.5.2. Time-dependent growth analysis

Using a growth kinetics experiment, the antibacterial effectiveness of PRP, M-IONP, and hydrogel on bacterial cell viability was assessed. To separate different bacterial colonies, the bacterial samples were first smeared onto nutrient agar plates. Ten mL of nutrient broth was inoculated with a single colony. The culture was then incubated overnight at 37 °C with constant shaking at 120 rpm. The optical density (1.0) of the culture at 600 nm was attained after incubation. The bacterial density was adjusted to 10^6^ CFU mL^−1^ by serially diluting the suspension across a 2-log range. After that, 20 μL of the bacterial suspension was exposed to different concentrations of M-IONPs and hydrogels in different wells of a 96-well microtiter plate. The wells were then incubated for 1 h at 37 °C. An Imark microplate reader from Biorad was used to measure optical density hourly at a wavelength of 600 nm.

#### 2.5.3. Cytoplasmic leakage analysis

*S. epidermidis*, *E. coli*, and *S. aureus* were cultivated in nutrient broth overnight to assess nucleic acid leakage in the cytoplasm. The next day, the pellet was obtained by centrifugation and proper washing. The pellets were resuspended in PBS buffer (pH 7.2). By keeping the bacterial cell count to 10^5^ cells per mL, these suspensions were treated with the hydrogel at 25 °C and incubated for 3 and 5 h. The hydrogels, which were not conjugated with the PRPs/M-IONPs, were used as controls. The cultures were centrifuged at 5000 rpm for 10 min, and the supernatant's absorbance was recorded at 260 nm to determine the concentration of nucleic acids in the cytoplasm.

#### 2.5.4. ROS determination assay

The amount of reactive oxygen species (ROS) produced when the hydrogel interacted with bacteria was measured utilizing 2′,7′-dichlorodihydrofluorescein diacetate (DCFH-DA). The bacterial cells were cultivated in a 96-well plate and treated with 100 μL of varied dilutions (25×, 50×, and 100×) of hydrogel for the ROS quantification assay. After 3 hours of incubation, 5 μL of 10 mM DCFH-DA stock solution was added. The fluorescence emission was detected at 523 nm with excitation at 503 nm using a Synergy H1 Microplate reader (Biotek, USA).

#### 2.5.5. Analysis of bacterial morphology using FE-SEM

Initially, sterile Sauton's medium was introduced into a 12-well plate containing bacterial inoculum of a 1 : 100 dilution of bacterial cells per well, which was treated with 100 mg of hydrogel. This setup was then incubated for 56 h to facilitate biofilm establishment. Following phenotypic observation, the biofilm-covered coverslips were retrieved and rinsed with distilled water and 1× PBS. Subsequently, 2.5% glutaraldehyde solution was added to the coverslips, followed by 20 min incubation at 4 °C to fix the adherent biofilms. After cleaning the coverslips with distilled water and 1× PBS, they were stained again for one hour at 4 °C using 1% osmium tetroxide. The coverslips were then washed with distilled water and 1× PBS, dried with varying concentrations of ethanol (30%, 50%, 70%, 90%, and 100%), and air-dried at room temperature. The slides were incubated overnight at 4 °C before undergoing SEM imaging.

### 2.6. *In vivo* wound healing study

#### 2.6.1. Creation of wounds

The role of hydrogels in the healing of the wound was assessed in a rat wound model. Female Albino Wister rats weighing 160–170 g were procured from the School of Pharmaceutical Sciences, Siksha O Anusandhan (Deemed to be University), Bhubaneshwar, Odisha, India. The rats were raised under standard conditions, maintaining a temperature of 25 °C and an approximately 12/12 h light–dark cycle, and were fed with the standard diet. Approval of the animal study protocol was obtained from the Institutional Animal Ethics Committee (IAEC), School of Pharmaceutical Sciences, Siksha O Anusandhan (Deemed to be University), Bhubaneshwar, with proposal number IEAC/SPS/SOA/185/2024.

The rats were kept in three groups: the control, standard, and hydrogel-treated groups. Each group contains 6 rats. Ketamine–xylazine (50–50 mg kg^−1^) was injected into the peritoneum of the rats to anesthetize them for wound creation in their dorsal skin and to implant a hydrogel wound dressing on the wound area. Dorsal skin was disinfected after shaving off the rats' back hair, and circular full-thickness wounds 1 cm in diameter were created on the dorsum of each rat. Then, as-prepared samples were spread on the wound's surface, and their effect was evaluated by measuring the wound area.

#### 2.6.2. Wound area

A high-resolution camera was utilized to capture wound region areas, and their analysis was performed using ImageJ software. For a period of 21 days, the wound area after wound induction was measured on alternate days. On day zero, surgery was performed. The wound reduction area was studied until complete wound healing. Finally, the percentage of wound closure was determined using the following equation:

4 Wound closure (%) = (*A*_0_ − *A*_*t*_)/*A*_0_ × 100,

where *A*_0_ is the wound area at the time of surgery and *A*_*t*_ is the area of the wound on day *t* post wounding.

## 3. Results

**Fig. 1:**
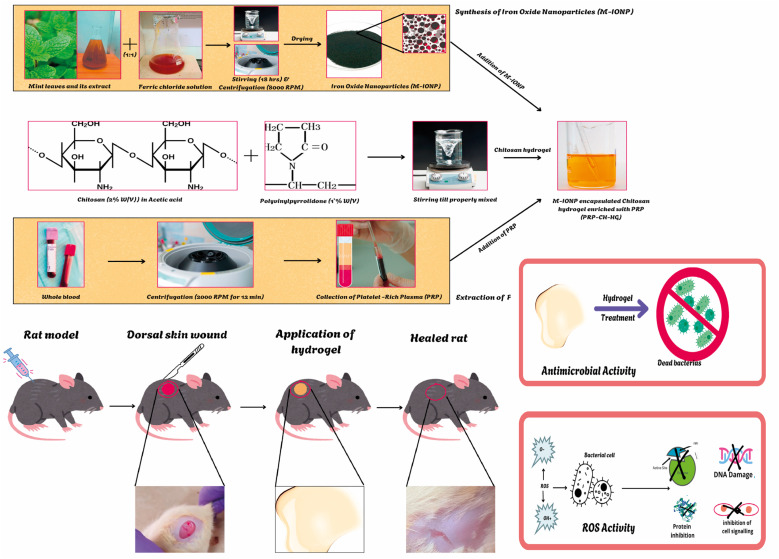
Schematic representation of PRP–CH–hydrogel preparation and its consequent wound healing application study.

The synthetic routes of gel preparation are illustrated in Fig. 1. Keeping in mind the clinical safety and easy translation, FDA-permitted chitosan and PRP were chosen for hydrogel synthesis. The hydrogel formulation was first studied by evaluating the dosage of M-IONP. From the antibacterial assays conducted, the optimal dose of the M-IONP was found to be 4 mg mL^−1^. The platelet concentration in PRP was measured using a Sysmex XP-300 automated hematology analyzer. The average platelet count was found to be 9.7 ± 0.2 × 10^5^ μL^−1^ (*n* = 3). These values confirm the efficiency of the Choukroun method for platelet concentration.^28^ PRP gel, which is extensively employed as an adjuvant in surgery, was developed by classic activation procedures. CaCl_2_ and thrombin were mixed with the PRP solution to aid in the coagulation of plasma and polymerization of fibrinogen into fibrin networks, leading to the synthesis of PRP gel. The gel preparation occurred as follows: at first, M-IONP was added to 30 mL of PVP/CS solution to obtain a homogenous mixture, followed by the addition of PRP gel. The procedure is a one-step synthesis as all the materials are added into one gelation medium to form networks. The prepared hydrogel exhibited an amorphous nature by conforming to the shape of the tube and could be injected *via* a syringe for application purposes when diluted. This amorphous nature of the hydrogel is crucial in wound management, as it enables the hydrogels to smear uniformly onto the wound and fill irregular defects. The bonds formed between iron oxide nanoparticles (M-IONPs) and chitosan (CS) typically involve a combination of electrostatic interactions, hydrogen bonding, and coordination bonds.^29^ Chitosan, being a cationic polymer, has amine (–NH_2_) and hydroxyl (–OH) functional groups that can interact with the surface of iron oxide nanoparticles, which often have a negatively charged surface due to hydroxyl groups (–OH) or oxygen atoms.^30^ The positively charged amine groups (–NH_3_^+^) of chitosan can electrostatically interact with the negatively charged surface of iron oxide nanoparticles, forming a stable bond.^30^ These interactions result in the stable attachment of chitosan to the iron oxide nanoparticles, enabling the formation of a robust hydrogel network with enhanced mechanical and functional properties. An investigation by applying a scanning electron microscope (SEM) showed a typical 3D structure with M-IONP in the interconnected pores appearing as flashy regions. The entire structure appears to be irregularly fused, giving the appearance of a matted structure primarily attributed to the low strength of fibrin. The sulfur element, the source of activated PRP detected in the hydrogel, demonstrated that the fibrin networks were embedded into the hydrogel. The morphological observations indicated that the hydrogels were successfully fabricated using the above synthesis method.

### 3.1. Characterization of M-IONP

#### 3.1.1. SPR analysis

**Fig. 2:**
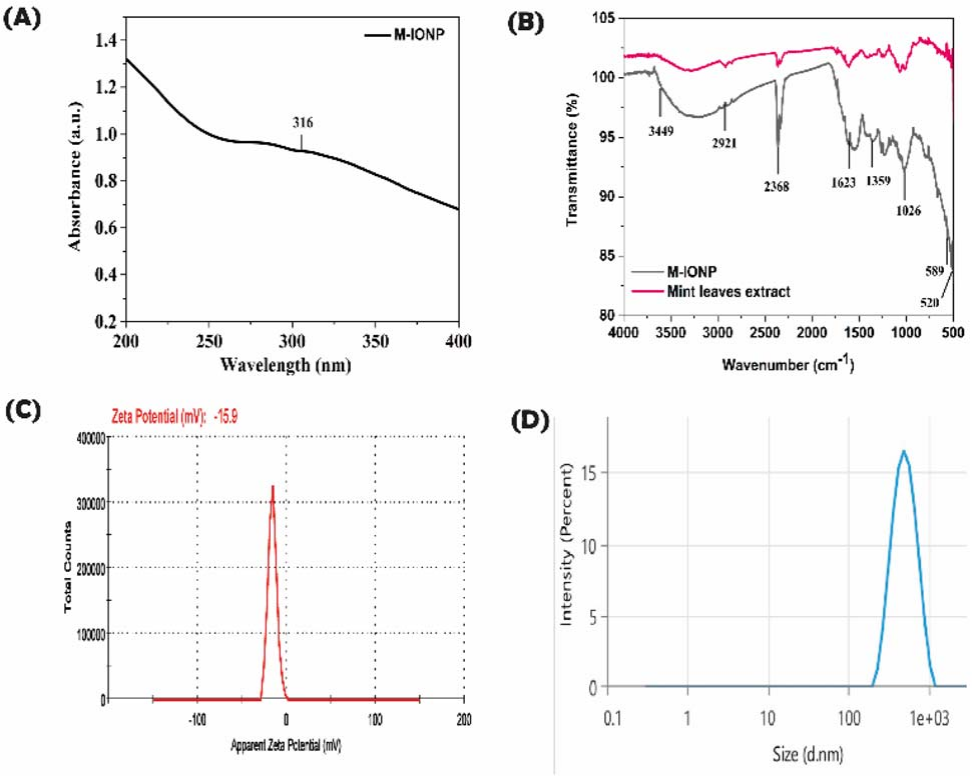
Characterization of the synthesized M-IONPs (synthesis conditions: pH = 9.2 and 25 °C): M-IONP's SPR peak was detected at 323 nm (A). Bond level characterizations of biosynthesized M-IONPs and mint leaf extract (B). Zeta potential of the M-IONPs (C) hydrodynamic size of M-IONPs (D).

After adding ferric chloride to the mint extract for eighteen hours, the M-IONP's SPR peak was detected at 323 nm (Fig. 2A). The production of mixed phases of iron oxides that eventually turn into pure magnetite is believed to be the reason why materials manufactured between pH 8 and 12 shift towards higher wavelengths, with a large absorption peak.^31^ Due to light scattering and absorption, magnetic particles tend to exhibit UV absorption peaks in the 300–450 nm range. Furthermore, the development of pure nanoparticles was demonstrated by the absence of additional absorption peaks in the UV-Vis spectra.^32^

#### 3.1.2. Functional group identification

Bond level characterization was conducted within the 400–4000 cm^−1^ range using FTIR for the identification of active components based on the peak value obtained (Fig. 2B). As shown in the figure, M-IONP synthesized from aqueous extracts of *M. Spicata* shows a peak in the regions: 3449 cm^−1^ corresponding to hydroxyl group stretching vibration.^33^ The spectral range between 3500 and 3200 cm^−1^ corresponds to the symmetric (sym) and asymmetric (asym) elongation of the polymeric hydroxyl group (O–H), as well as hydrogen bonding, indicating the presence of specific functional groups. Notably, these groups are bonds, like C–H, C–O, C–N, and P–O.^34^ The 2921 cm^−1^ and 2368 cm^−1^ peaks are identified with the symmetrical stretching of the sp^3^ carbon.^33^ The 1623 cm^−1^ band is related to the bending node of water, indicating the strong affinity of the plant extract for water.^33^ 1359 cm^−1^ denotes C=C stretching. The peak at 599 cm^−1^ correlates to aromatic H out-of-plane bending.^33^

589 and 520 cm^−1^ observed peaks are related to magnetite, resulting from the Fe–O–Fe bonds present in the compound.^35^ Additionally, the peaks in the 500–600 region denote the metal–metal interactions.^36^ The FT-IR data further confirm that bioactive molecules, like amines, phenols, carbonyls, and alkenes, present in the leaf extract of *M. spicata*, play a significant role as reducing agents in the synthesis of M-IONP.^37,38^

#### 3.1.3. Hydrodynamic size and surface potential analysis

The zeta potential of M-IONPs was found to be −15.09 (Fig. 2C). Similarly, the hydrodynamic size of M-IONP was found to be 485.2 nm (Fig. 2D). The *x*-axis represents particle size on a logarithmic scale (0.1–1000 nm), with the peak indicating a dominant particle size population around ∼310 nm. The *y*-axis shows the relative intensity percentage of the measured size range.

#### 3.1.4. Morphological analysis

**Fig. 3:**
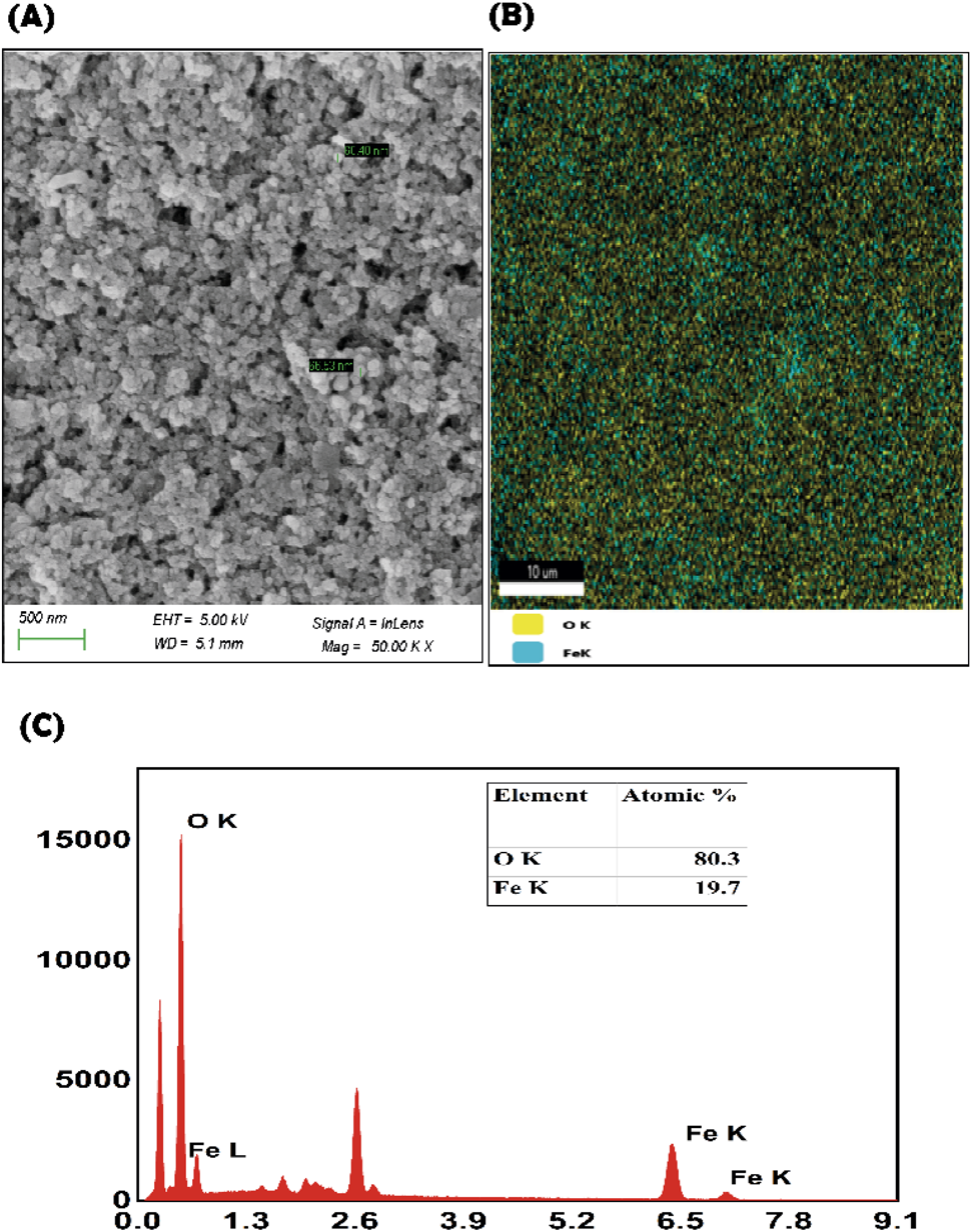
Morphological analysis of M-IONPs (A), elemental analysis of M-IONPs (B) and EDAX analysis of M-IONPs (C).

The green synthesized M-IONP indicates the formation of spherical-shaped M-IONPs (Fig. 3A). The surface morphology, as indicated by the images, is smooth, and the sizes range from 80 to 100 nm. The *M. spicata*-mediated M-IONPs are composed of 80.03% of iron and 19.7% of oxygen (Fig. 3B). The EDAX results represent the elemental composition of the synthesized M-IONP (Fig. 3C).

### 3.2. Characterization of PRP–CH–hydrogel

#### 3.2.1. Morphological and elemental analyses

**Fig. 4:**
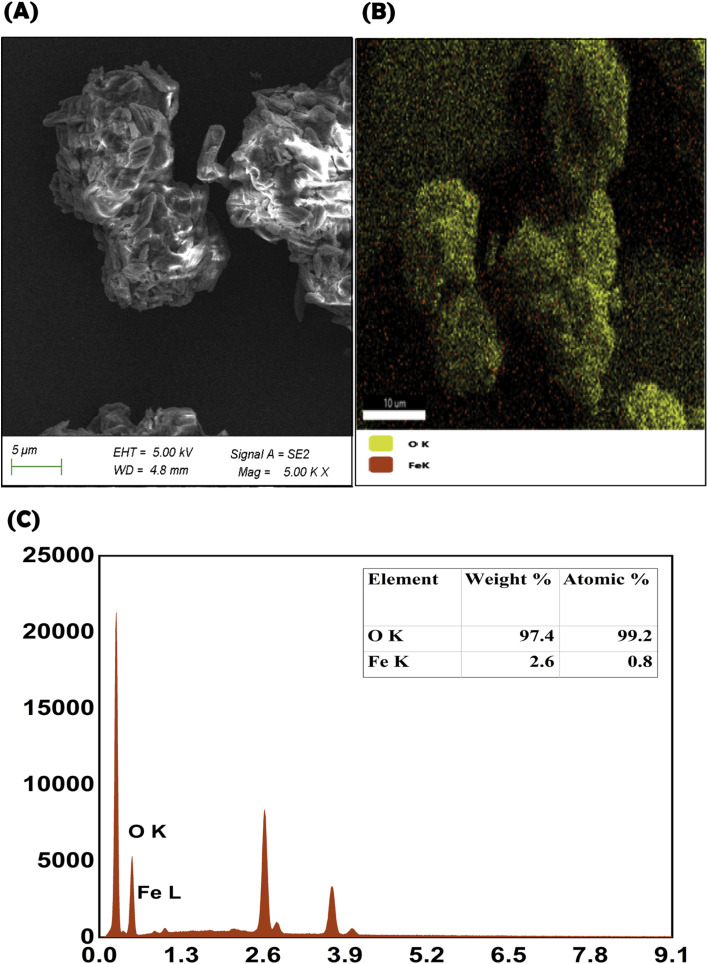
SEM micrograph of the PRP–CH–HG (A). Elemental (B) and EDAX (C) analyses of the PRP–CH–HG.

The SEM micrograph of the PRP–CH–HG demonstrated the surface morphology and distribution of the green synthesized nanoparticles embedded within the matrix (Fig. 4A). The nanoparticles appeared bright, exhibited irregular regions, and were uniformly distributed across the scaffold. The hydrogel exhibited a textured and irregularly aggregated porous matrix. The porous matrix indicated the facile and effective integration of the iron oxide nanoparticles and the PRP into the scaffold structure. The elemental composition of the hydrogel spectra was validated at the Kα P signal at 2.02 keV, confirming the successful development of PRP and nanoparticle-incorporated hydrogel. The elements C, O, Na, Cl, Ca, and Fe were identified (Fig. 4B and C).

#### 3.2.2. Functional group identification

**Fig. 5:**
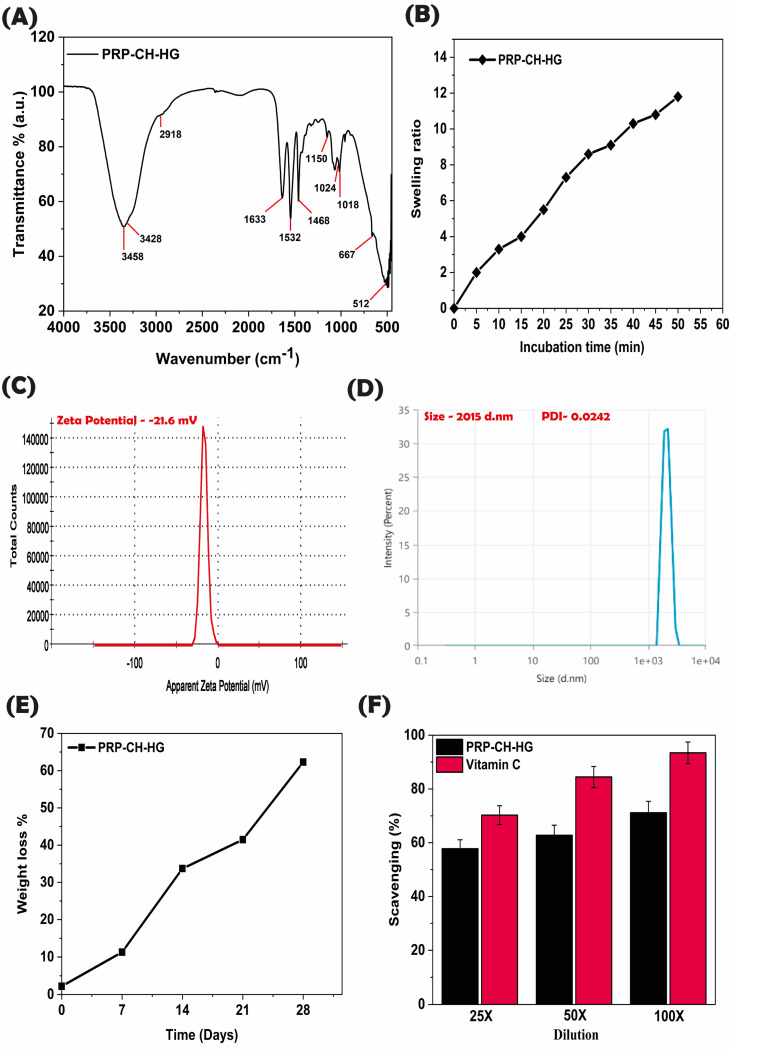
FT-IR spectroscopic analysis of the PRP-conjugated iron-doped chitosan hydrogel (A). Swelling ratio of the hydrogel (B). Zeta potential of the PRP–CH–HG (C). Hydrodynamic size of the PRP–CH–HG (D). Hydrogel degradation rate (E); number of replicates (*n* = 3). Antioxidant property of hydrogel (F); number of replicates (*n* = 3).

The FT-IR spectroscopic analysis revealed the formation of PRP-conjugated iron-doped chitosan hydrogel (Fig. 5A). Conventional chitosan signals that characterized the spectra are as follows:

3428 cm^−1^–3458 cm^−1^: the broad peak between 3500 and 3250 cm^−1^ is due to the O–H stretching of inter- and intramolecular hydrogen bonding.^39^

2918 cm^−1^: the peak at 2950–2800 cm^−1^ is the typical C–H stretch vibrations.^39^

1633 cm^−1^: the amide I linkage peaks.^39^

1532 cm^−1^: the amide II linkage peaks.^39^

1468 cm^−1^: the new bands in the spectrum at 1468 cm^−1^ are due to asymmetrical and symmetrical deformation modes of COO^−^, which confirm the existence of acrylic acid.^39^

1150 cm^−1^: the characteristic peak of the saccharine structure.^39^ The appearance of new bands associated with the interaction of metal ions confirms the successful doping of iron oxide nanoparticles. The bands at 512 cm^−1^ correspond to metal–oxygen bonding in the case of nanoparticles. Additionally, the FTIR spectroscopic data confirmed the nanoparticles and PRP loading on the chitosan hydrogel.

#### 3.2.3. Hydrodynamic size and charge analysis

The zeta potential of PRP–CH–HG was found to be −21.06 (Fig. 5C). Similarly, the hydrodynamic size of PRP–CH–HG was found to be 2015 nm (Fig. 5D). The conjugation of PRP and chitosan moieties increased the hydrodynamic size.

### 3.3. Property analysis of the hydrogel

#### 3.3.1. Swelling of hydrogel

In the case of an ideal wound dressing, the maximum absorption of exudates should take place at the wound site, along with the inhibition of bacterial growth. The stages involved in the hydrogel comprise water intrusion into the polymer network, polymer chain relaxation, and polymer network expansion. Fig. 5B depicts the swelling ratio of the hydrogel upon incubating for 50 min, which was found to be 12.87 ± 0.25. The presence of chitosan and PRP in the hydrogel hindered the swelling behavior primarily because chitosan has a relatively lower porosity. Moreover, the hydrogen bond formation between the nanoparticles and the hydrogel may lower the swelling capacity. Similarly, studies have revealed that CH nanoparticles in the core of the CH-PRF scaffold lead to poor swelling behavior.

#### 3.3.2. Spreadability of hydrogel

The spreadability of the chitosan-based hydrogel containing PVP was found to be 272 ± 0.10 g cm min^−1^, which was slightly higher than that of the control-only chitosan gel (268 ± 0.02 g cm min^−1^). The enhanced spreadability may be attributed to the hydrophilic nature of PVP, which improves the gel's ability to deform under applied pressure and ensures better surface coverage.

#### 3.3.3. Degradation assay

The hydrogel degradation rate is depicted in (Fig. 5E). The weight loss percentage of the hydrogel was determined to be 62% ± 03% after 28 days. The hydrogen bonding between the nanoparticles and the hydrogel resulted in a decreased degradation rate compared to the free hydrogel. This result is supported by a similar study showing that the degradation process of GelCH hydrogel/nanofibrin decreased and that the fibrin present in the composite strengthened the wound dressing.^40^

#### 3.3.4. Free radical scavenging assay

The hydrogel's antioxidant properties should inhibit bacterial growth in the wound area. This study focuses on evaluating the ability of hydrogel to scavenge free radicals *via* a DPPH assay (Fig. 5F). The scavenging activity of the hydrogel was measured to be 57.75%, 62.75%, and 80.28% for 25×, 50×, and 100×, respectively. The hydrogel's antioxidant activity was observed in a concentration-dependent manner. The presence of M-IONP and CH in the hydrogel heightened the antioxidant activity of the hydrogel. The amine, hydroxyl groups, and ammonium cations (–NH^3+^) provided by chitosan impart antioxidant properties to the hydrogels.^41^ Thus, the amination mechanism and the electron-donating ability of CH enhance the free radical scavenging activity of the hydrogel.

### 3.4. Assessment of *in vitro* biocompatibility

#### 3.4.1. Biocompatibility assay

**Fig. 6:**
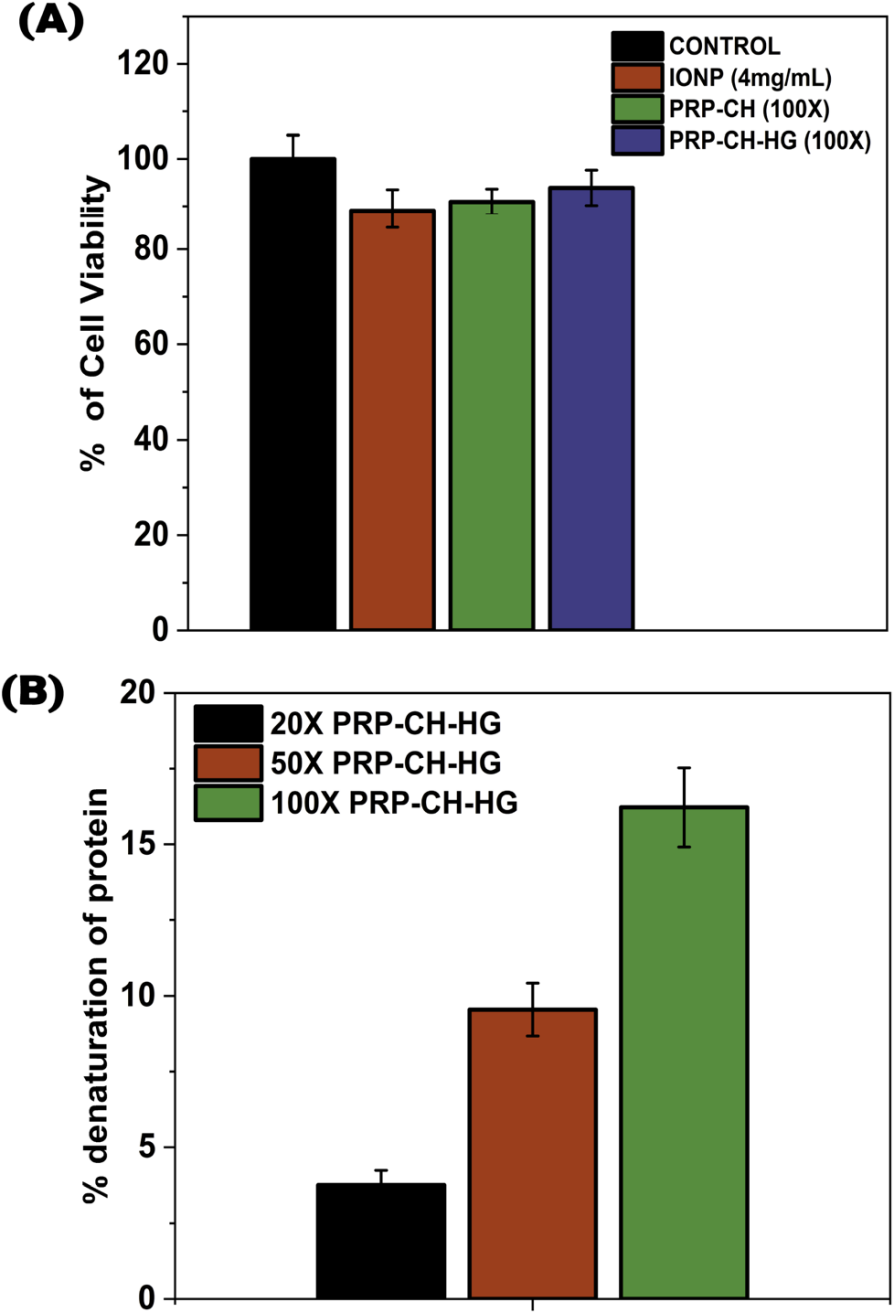
MTT assay study demonstrating that the hydrogel is biocompatible (A). Anti-inflammatory assay (B).

MTT assay was utilized to study the HEK cell viability upon treatment with the hydrogel samples (Fig. 6A). The cell viability for the hydrogel was 93.03% at 100× concentration. There was no significant difference between the hydrogel and control sample (99%). There was an enhancement in the cell viability at the surface of the hydrogel owing to its increased porosity and good specific surface area. PRP present in the hydrogel played a significant role in increasing cell viability compared to the other chitosan-based hydrogels. Additionally, CH, being a polycation, increases the hydrophilicity and hence changes the surface charge of the hydrogels, resulting in increased interactions with the cells of the affected area, thereby facilitating an ambient environment for cell growth and proliferation.^42,43^ The availability of the arginine–glycine aspartic (RGD) sites on the hydrogel's chains allows the integrin proteins of the cells to interact with the hydrogels, which increases cell proliferation and cell viability.^44^ Additionally, the regulated release of growth factors and cytokines from PRP enhances the viability and proliferation of cells.^45,46^ Thus, the *in vitro* MTT assay study demonstrates that the hydrogel is biocompatible and can offer an excellent environment for the proliferation of cells.

#### 3.4.2. Anti-inflammatory assay

The anti-inflammatory assay demonstrates that PRP–CH–HG inhibits protein denaturation in a volume-dependent manner (Fig. 6B). Preventing protein denaturation is critical in mitigating inflammatory responses, as denatured proteins often act as triggers for immune activation. This property positions PRP–CH–HG as a candidate for therapeutic use in inflammation-associated diseases, such as arthritis or autoimmune disorders. Comparative analysis with standard anti-inflammatory drugs, like diclofenac, would strengthen these claims. Additionally, molecular docking studies could help identify the specific interactions between PRP–CH–HG and protein targets.

#### 3.4.3. Hemolysis assay

**Fig. 7:**
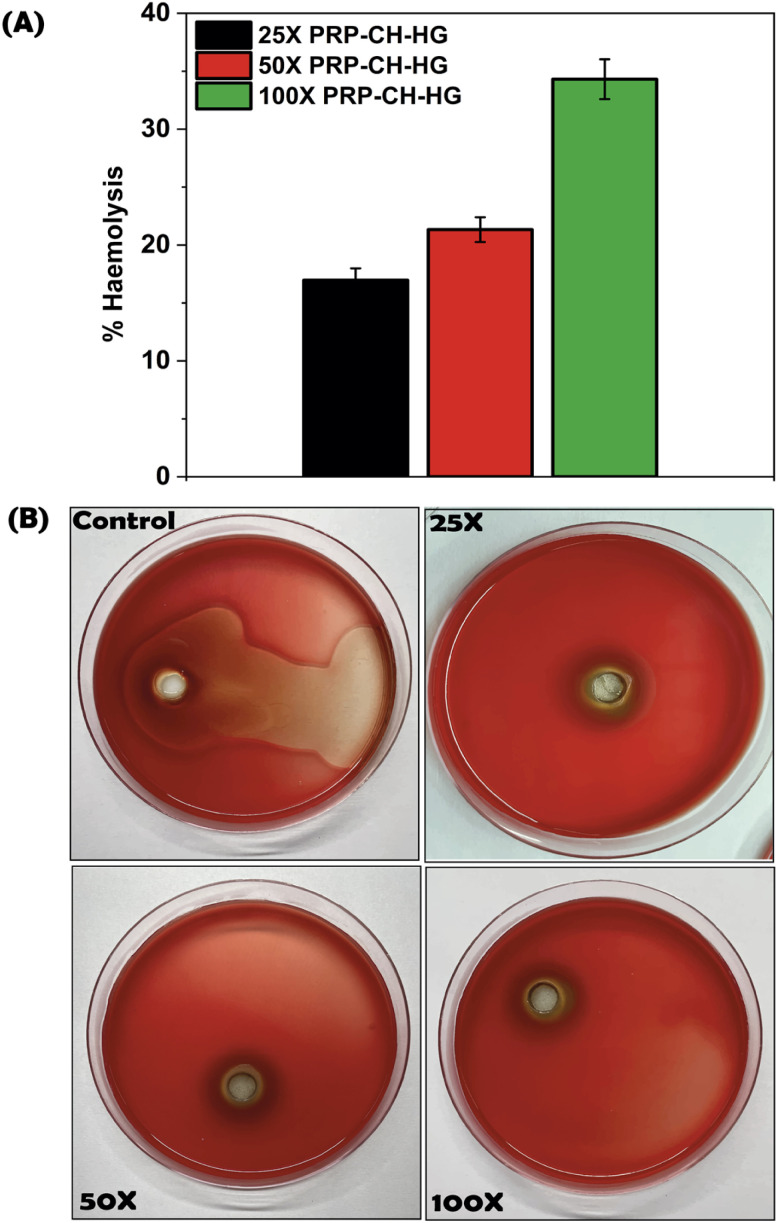
Hemolysis data showing a concentration-dependent increase in red blood cell lysis by the PRP–CH–HG (A) and (B).

The hemolysis data show a concentration-dependent increase in red blood cell lysis by PRP–CH–HG, with hemolysis levels increasing from approximately 20% at 25× to 33% at 100× (Fig. 7A and B). Although this indicates some cytotoxicity, hemolysis levels below 50% are generally considered safe for biomedical applications. Optimization of concentration is critical to ensure efficacy while minimizing adverse effects. The hemolytic activity may stem from interactions between PRP–CH–HG and the lipid bilayer of red blood cells potentially due to surface charge or hydrophobic interactions. In localized delivery applications, the formulation is administered directly to the target site, thereby limiting its interaction with circulating erythrocytes and minimizing systemic exposure. Under such conditions, the hemolysis observed in our assays is less likely to translate into adverse *in vivo* effects. This context reinforces the safety of the system for site-specific therapeutic interventions while clarifying that hemocompatibility considerations require more stringent evaluation for potential systemic administration. Further investigations into these mechanisms using techniques, like membrane stability assays or surface charge analysis, would provide deeper insights.

### 3.5. Antibacterial activity of the hydrogel

#### 3.5.1. Time-dependent kinetics

**Fig. 8:**
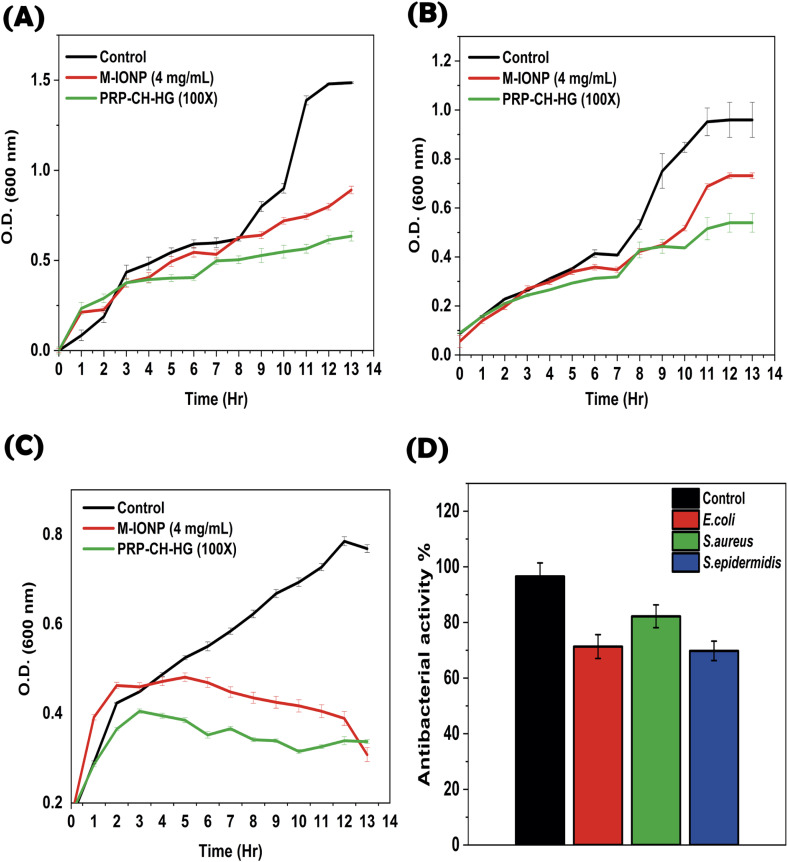
Growth curves for the selected microorganisms: *S. epidermidis* (A), *S. aureus* (B), and *E. coli* (C). The histogram shown in (D) illustrates the antibacterial activity of the hydrogel.

Antibiotic resistance is the main concern for researchers investigating new biomaterials that have effective wound healing properties with minimal infection risk. A comparative analysis was performed to evaluate the enhanced antimicrobial effects of each compound and the incorporation of these compounds as hydrogels. As depicted in Fig. 8, the efficacy of the effective dose and killing time for the hydrogel and M-IONP against the three tested pathogenic microorganisms was examined independently. Overall, these results indicated that a combination of these compounds altogether in a hydrogel framework synergistically improves its antibacterial properties. Additionally, anchoring of M-IONPs significantly boosted antimicrobial activity, enhancing its effectiveness against such pathogenic microorganisms. Incorporating M-IONPs into the formulation significantly augmented their antimicrobial activity through classical nanomaterial-ROS-mediated killing mechanisms. Customarily, the bacterial growth kinetics curve, depicted as a growth curve, tracked variations in bacterial population size over time. The growth curve, a bacterial population dynamics model, consists of four phases: lag, logarithmic, stationary, and death. It is crucial for understanding microbial dynamics, assessing growth impacts, and optimizing conditions. Fig. 8A–C depict the growth curves for *S epidermidis*, *S. aureus*, and *E. coli*, respectively. Notably, the initial growth curve of these microbes showed a time-dependent delay after inactivation with both M-IONP and PRP–CS–HG. This implies that both M-IONP and PRP–CS–HG were effective in progressively hindering the growth of these microbes. It is crucial to observe that the efficacy of both M-IONP and PRP–CS–HG against microbial growth might vary with microbial strains and environmental conditions. These observations were attributed to the greater efficacy of the hydrogel against Gram-negative over Gram-positive bacterial species. The reason might be that the invulnerable architecture of Gram-positive bacteria over Gram-negative bacteria was ascribed to the highly robust and compact peptidoglycan, consisting of the cell wall.^47^ Notably, the Gram-negative strain is more sensitive to both M-IONP and PRP–CS–HG than the other strains, which is consistent with previous studies. Regarding the lethal doses of both M-IONP and PRP–CS–HG, exposure to a lethal dose of 100× PRP–CH–HG and 4 mg mL^−1^ of M-IONP significantly reduced bacterial growth across all tested species. Consequently, further studies are warranted to assess the safety and efficacy of the hydrogel for specific applications.

**Fig. 9:**
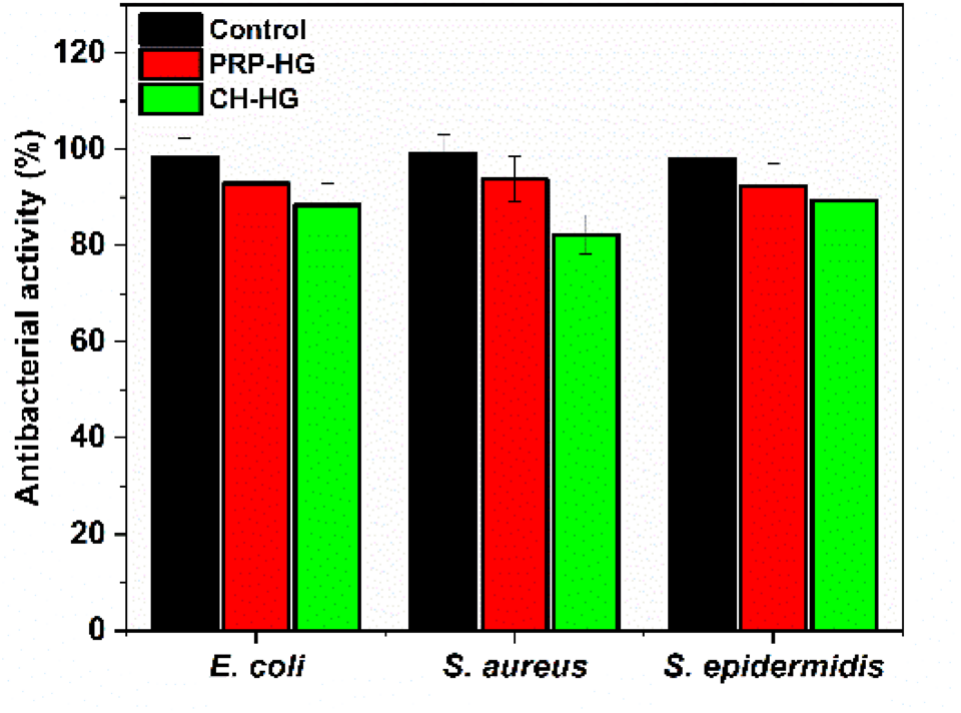
Comparative antibacterial activity of the PRP–HG and CH–HG.

The results ascertained the enhanced bacterial killing property of the PRP–CH hydrogel, reflecting the potential of chitosan and M-IONP to serve as an intrinsic antibacterial agent. The CH particles also play a role in enhancing the wound-healing process. The histogram shown in (Fig. 8D) illustrates the antibacterial activity of the hydrogel on *E. coli*, *S. aureus*, and *S. epidermidis*, which was 71.33%, 82.22%, and 69.78%, respectively. This result is in agreement with the earlier report by Xu *et al.*, showing the high antibacterial activity of a scaffold based on decellularized extracellular matrix/CH/Gel due to the presence of CH (as it preserves moisture) in the scaffold.^48^ Another study by Tamer *et al.* demonstrated the antibacterial properties of *O*-amine-functionalized CH against four bacterial strains, showing the high antibacterial activity of CH.^49^ Additionally, a comparative antimicrobial activity evaluation was carried out for PRP–HG and CH–HG to highlight individual activity and their synergistic potential (Fig. 9). CH–HG, which has only chitosan, shows a mild-moderate antimicrobial effect, especially against Gram-positive bacteria. PRP–HG showed the least antimicrobial activity. These results throw light on the hydrogel's ability to nullify the infection caused by Gram-positive and Gram-negative bacteria.

#### 3.5.2. Leakage of nucleic acids

**Fig. 10:**
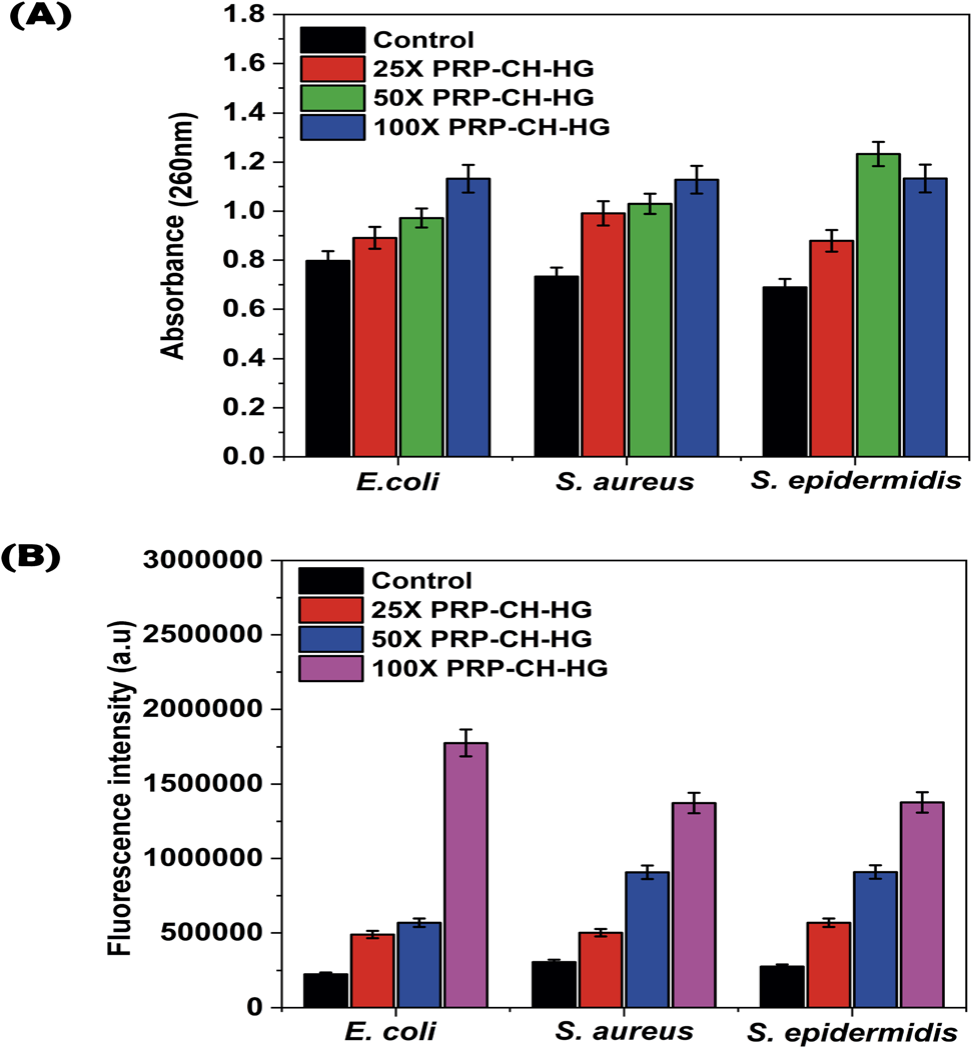
Nucleic acid leakage from a microbial cell (A). Fluorescence intensities across different hydrogel dilutions (B).

Nucleic acid leakage from microbial cell membranes is a common issue resulting from membrane damage or disruption often linked to antimicrobial agents, physical stress, or environmental conditions (Fig. 10A). The figure represents the proportional release of nucleic acid from the bacterial cell membranes of the tested microorganisms. The results depicted in Fig. 10A revealed a significant increase in the nucleic acid released from bacterial cells when exposed to effective dosages of hydrogel at 25×, 50× and 100×. This suggests that the tested compounds likely impacted bacterial cell membrane permeability, contributing to their inhibitory effects on bacterial growth and proliferation. This implies that these compounds can substantially influence microbial membrane integrity, resulting in heightened permeability and intracellular constituent leakage.

#### 3.5.3. ROS analysis

The fluorescence intensities, which indicate the level of ROS production, varied significantly across the different hydrogel dilutions (Fig. 10B). A 25× dilution showed a moderate increase in fluorescence intensity, indicating a moderate ROS generation level. Similarly, the 50× dilution demonstrated a significant increase in fluorescence intensity compared to the 25× group, suggesting higher ROS production. Subsequently, 100× dilution displayed the highest fluorescence intensity, indicating the maximum level of ROS generation among all tested concentrations. The results revealed a dose-dependent increase in ROS production, with higher hydrogel concentrations promoting elevated ROS levels. This indicates the potential of the hydrogel to induce oxidative stress in bacterial cells, contributing to its antimicrobial mechanism of action.

#### 3.5.4. Morphological SEM observation

**Fig. 11:**
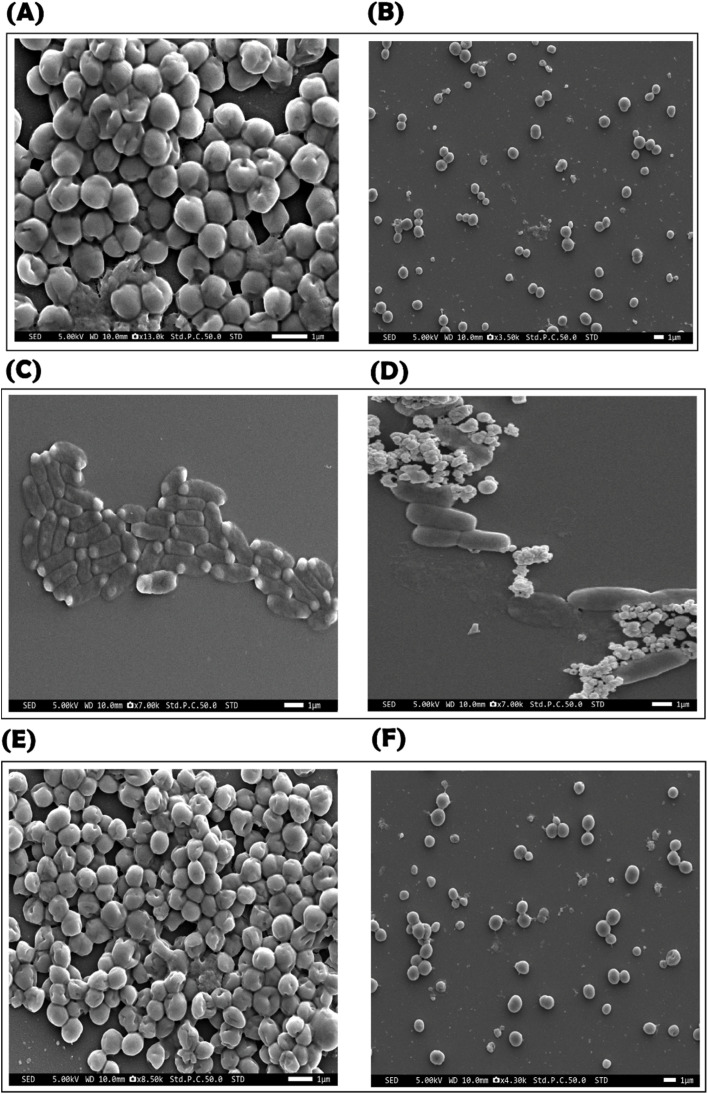
Morphological effects of the hydrogel on the surfaces of *S. epidermidis* (control (A); treatment (B)), *E. coli* (control (C); treatment (D)), and *S. aureus* (control (E); treatment (F)).

SEM imaging was conducted to evaluate the effect of the hydrogel on the morphology of *S. epidermidis*, *E. coli*, and *S. aureus* (Fig. 11). Herein, bacterial cells were exposed to a 100× concentration of the hydrogel (without dilution) compared to untreated control samples, followed by SEM analysis. The SEM images revealed distinct morphological alterations in the bacterial surfaces after hydrogel treatment. In untreated bacterial samples, *E. coli* exhibited intact (Fig. 11C), rod-shaped cells, while *S. epidermidis* (Fig. 11A) and *S. aureus* (Fig. 11E) maintained their characteristic spherical morphology and dense biofilm structure in the control slides. Upon hydrogel treatment, significant structural damage was observed in the bacterial species. The treated *E. coli* cells displayed wrinkled and collapsed surfaces, while *S. epidermidis* and *S. aureus* showed disrupted cellular clusters and surface deformation. Moreover, there was a noticeable reduction in bacterial population density compared to the control samples. The hydrogel appeared to induce cell lysis and hinder biofilm formation, contributing to the bacterial population decline. These findings suggest that the hydrogel effectively compromises bacterial membrane integrity and disrupts biofilm structures, highlighting its strong antimicrobial activity.

#### 3.5.5. Wound healing

**Fig. 12:**
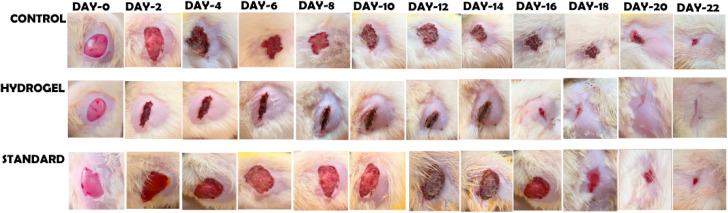
Wound healing activity in the rat model exhibited by the PRP–CH–HG.

On the initial day (day 0), all rats presented wounds of uniform size. Gradual wound closure was observed in all groups, with the most significant reduction in wound size detected during the first 8 days (Fig. 12). By day 14, over 70% of the wound area had closed in the case of the hydrogel-treated rats. Complete wound healing (≥98% wound closure) was achieved by day 18 in the hydrogel-treated animals. Complete wound healing in the control and standard-treated rats began on day 22.

The data revealed statistically significant differences between the experimental and control groups, indicating enhanced wound healing in the hydrogel treatment group(s). The graphical representation of wound closure percentages over 21 days demonstrates the accelerated healing process in the treated group compared to the untreated control group. This study's results suggest that the treatment effectively promoted faster and more efficient wound healing compared to untreated rats, supporting its potential application in wound management.

## 4. Discussion

Until the late 1950s, the belief prevailed that maintaining a dry environment for wounds was essential for their healing and the prevention of bacterial infection. Dry dressings promote healing by keeping the wound site dry until a crust is formed. However, numerous studies have challenged this approach, revealing that moist wounds heal significantly faster than those exposed to dry dressings/air. In a review of occlusive dressing and wound healing, Winter *et al.* reported that maintaining wound moisture could enhance the rate of epidermal resurfacing by approximately 40%.^50,51^ Wet dressing in contrast to dry dressings creates a moist environment that facilitates the movement of epithelial cells, leading to faster healing, reducing dry necrosis and providing better wound protection. Hydrogels resemble molecular sponges that have intricate networks with a dispersion medium trapped within their structure.^52^ These hydrogels are generally composed of insoluble polymers with a hydrophilic region that facilitates interaction with water, enabling them to absorb and retain large quantities of fluids.^52^ In the current scenario, with the increased use of hydrogels in various biomedical applications, such as drug delivery platforms, wound healing and tissue scaffolding materials, and membrane filtration devices, it is of great importance to understand the behavior of the polymer network.

Chitosan, a copolymer of glucosamine and *N*-acetyglucosamine units linked by 1–4 glucoside bonds, is obtained by *N*-deacetylation of chitin. It is one of the most abundant natural amino polysaccharides favored widely owing to its strong interaction with polar and charged molecules. It can react with negatively charged molecules, forming a three-dimensional network through ionic bridges between polymer chains. This partially deacetylated form of chitin is a material widely used presently in the wound management field owing to its acclaimed hemostatic properties. In general, chitosan with a high molecular weight is not soluble in water but dissolves in an acid-water solution. In fact, as a necessary property, hydrogels must undergo volume change, remain insoluble, and exhibit stable strength. Therefore, the present study exploited chitosan as the hydrogel base and PVP as a crosslinker by employing a moist heat treatment technique for the formation of the hydrogel. As extensively reported in the literature, chitosan reacts with polyvinylpyrrolidone (PVP), and the mechanism of gelation shifts from cross-linking to hydrogen bonding and physical interaction owing to the neutral charge of PVP. The hydrophilic groups of PVP lead to the formation of hydrogen bonds, creating a three-dimensional network through non-covalent forces. The presence of PVP enhances the gel's water retention capacity and modifies its mechanical properties since the hydrophilic nature of PVP allows its smooth integration into the matrix of chitosan. The PVP–chitosan blend hydrogel shows blood compatibility.^53^ The hydrogels were developed by applying a physical stimulation technique, which is an operation-friendly technique. Hydrogels, nanomaterials, and peptides can serve as effective treatment methods to tackle chronic skin wounds when combined. Fu, Z. *et al.* reported a combination of Cy_RL–QN15_ peptide, sodium alginate hydrogel, and polydopamine nanoparticles (HPDAlCy_RL–QN15_/ZA hydrogel), which showed the healing of full-thickness skin wounds in type2 diabetic mice.^54^ Cy_RL–QN15_ peptides have also been shown to promote hair regeneration in a diabetic skin model, which accelerates the healing of diabetic skin damage. Cy_RL–QN15_ peptides were also reported to inhibit miRNA-365-2-5p at the wound site in mice, thereby increasing SIRT1 and FOXO1 protein expression and decreasing STAT protein expression, leading to overall cell proliferation and wound healing.

Another common problem impeding wound healing is drug-resistant bacterial infection and excessive inflammation, which highlights the necessity of developing multifunctional pro-healing drug candidates. Li X. *et al.* reported a nanoparticle system combining curcumin (CCM), an aggregation-induced emission luminogen (TTD) and ZIF-8 (CCM + TTD@ZIF-8 NPs), which showed efficient bactericidal activity against drug-resistant *Staphylococcus aureus* (*S. aureus*) and *Pseudomonas aeruginosa* (*P. aeruginosa*), both *in vitro* and *in vivo*.^55^ Jia Q. *et al.* reported a complex photothermal antibacterial composite hydrogel by employing carboxymethyl chitosan (FeCMCS), melanin nanoparticle (MNP), and Cy_RL–QN15_ peptide (MNP/Cy_RL–QN15_/FeCMCS), which enhanced keratinocyte and fibroblast growth and elicited enhanced antibacterial activity.^56^ Recent advancements have also been made to develop inorganic-based biomaterials, such as carbon dots, metal oxides, hydroxyapatites, and silicate-containing composites, because they have the potential to significantly improve the physical, chemical, and biological properties of skin substitutes.^57^

Biomolecules and nanoparticles can be immobilized on and within hydrogels to make them suitable for specific purposes. Molecules and cells immobilized on or within hydrogels are more likely to retain their biological activity for a longer time. The SEM image showed that the M-IONPs provided the hydrogel with more structural reinforcement because the PRP encapsulation within the hydrogel network affects its cohesiveness.

## 5. Conclusions

The successful development of a smart wound-healing hydrogel using biosynthesized iron oxide nanoparticles (M-IONPs) to enhance antibacterial activity and encourage quicker wound healing was highlighted in this study. The hydrogel demonstrated regulated PRP protein production, which successfully reduced bacterial infections and promoted tissue repair. According to TEM-based morphological alterations, the addition of green-synthesized M-IONPs greatly increased antibacterial efficiency, especially against Gram-negative bacteria. Furthermore, in an animal model, chitosan-based hydrogel showed remarkable effects on wound healing, including complete healing in 18 days, high cell survival, and great biocompatibility. These results highlight the potential of organic-inorganic hybrid hydrogels with surface functionalization as novel and efficient therapeutic platforms for the treatment of infected wounds, providing a promising strategy for upcoming biomedical applications.

## Author contributions

Lipsa Leena Panigrahi: conceptualization, investigation, methodology, validation, writing-original draft; Siddharth Satapathy: data curation; Pallavi Samal: investigation; Shashank Shekhar: data curation; and Manoranjan Arakha: conceptualization, formal analysis, methodology, supervision, review and editing.

## Conflicts of interest

There are no conflicts to declare.

## Data Availability

All data described in this study are contained within the main article.
